# Draft Genome Sequence of *Rhodococcus* sp. Strain ATCC 49988, a Quinoline-Degrading Bacterium

**DOI:** 10.1128/MRA.00403-19

**Published:** 2019-06-20

**Authors:** Nidhi Gupta, Kelly A. Skinner, Zarath M. Summers, Janaka N. Edirisinghe, José P. Faria, Christopher W. Marshall, Anukriti Sharma, Neil R. Gottel, Jack A. Gilbert, Christopher S. Henry, Edward J. O’Loughlin

**Affiliations:** a Argonne National Lab (ANL), Argonne, Illinois, USA; b University of Chicago, Chicago, Illinois, USA; c ExxonMobil Research and Engineering Company, Annandale, New Jersey, USA; University of Maryland School of Medicine

## Abstract

We report here the 4.9-Mb genome sequence of a quinoline-degrading bacterium, *Rhodococcus* sp. strain ATCC 49988. The draft genome data will enable the identification of genes and future genetic modification to enhance traits relevant to heteroaromatic compound degradation.

## ANNOUNCEMENT

Quinoline is a nitrogen-containing heterocyclic compound that is used as a solvent in the chemical industry and occurs widely in coal tar, oil shale, and plant alkaloids. It is known to be a mutagenic and carcinogenic compound (1). Due to its widespread use and toxic properties, the degradation of quinoline by microorganisms has been studied extensively. *Rhodococcus* sp. strain ATCC 49988 was isolated from soil by enrichment culture using quinoline as a dominant carbon, nitrogen, and energy source (2). In order to understand the genetic basis of quinoline degradation and other abilities of this strain, genome sequence analysis of *Rhodococcus* sp. strain ATCC 49988 was carried out.

*Rhodococcus* sp. strain ATCC 49988 was obtained from the ATCC. It was grown in tryptic soy broth at 30°C with shaking at 200 rpm for 16 h, after which DNA for whole-genome sequencing was isolated using a DNeasy PowerSoil kit (catalog number 12888-50; Qiagen), followed by preparation of an Illumina Nextera XT paired-end library. Whole-genome sequencing was performed using a MiSeq system (Illumina) and produced 13,615,724 (average length, 160 bp) paired-end reads. The raw sequence was processed using the KBase (3) platform (https://narrative.kbase.us/narrative/ws.39490.obj.1), and the sequence was uploaded in FASTQ format. During analysis, default parameters were used for all software, unless otherwise specified. The read quality was assessed using FastQC v 0.11.5 (4), and the adaptor sequences specific to the Nextera DNA library were trimmed using Trimmomatic v 0.36 (5). *De novo* assembly conducted using SPAdes v 3.12.0 (6) yielded 27 contigs. The draft genome sequence has a total length of 4,970,306 bp (*N*_50_, 792,857 bp) and a GC content of 67.87%. The genome coverage was 155.0×. Genome assembly statistics were computed using QUAST v 0.0.4 (7). The assembled contigs ranged in size from 805 bp to 1,052,617 bp. The assembly was evaluated for completeness and contamination using CheckM v 1.0.8 (8), which characterized the assembly as 99.4% complete with 0% contamination.

The genome sequence of this *Rhodococcus* sp. strain was first annotated using Prokka v 1.12 (9), which predicted a total of 4,508 protein-coding genes in the genome (https://narrative.kbase.us/#dataview/39490/15/1). Next, the genome was functionally annotated using Rapid Annotations using Subsystems Technology (RAST) v 0.1.1 (10; https://narrative.kbase.us/#dataview/39490/17/1), which assigned 1,406 (31%) of the genes to SEED subsystems. Several putative polycyclic aromatic hydrocarbon (PAH) catabolic enzymes (11), such as monooxygenase, dioxygenases, dehydrogenases, and cytochrome P450, were found in the *Rhodococcus* genome. However, genes for quinoline 2-oxidoreductase were not annotated in the genome, indicating that this species uses a different enzyme to convert quinoline to 2-hydroxyquinoline. The genomic information of this *Rhodococcus* sp. strain will not only facilitate our understanding of the metabolism for recalcitrant heteroaromatic compounds but will also expand our knowledge of the physiology of the *Rhodococcus* genus.

### Data availability.

The draft genome sequence of this *Rhodococcus* sp. strain has been deposited in GenBank under the accession numbers SDMJ01000001 to SDMJ01000026, and the raw sequencing reads are available in the Sequence Read Archive under accession number SRP179964.
